# The Transactions of the Provincial Medical, and Surgical Association

**Published:** 1839

**Authors:** 


### THE TRANSACTIONS OF THE PROVINCIAL MEDICAL, AND SURGICAL ASSOCIATION. Vol. VII. London. Churchill, 1839.

ANOTHER volume of the Provincial Society's Transactions is before us. It furnishes ample evidence of the zeal, the unanimity, the information, and the talent of a large body of our provincial brethren. Long may they combine for these worthy ends—the promotion of union among themselves, and the diffusion of science in the empire.

The contents of the volume are the following:—

1. Proceedings of the Association at Bath. 2. The Retrospective Address, delivered at the Sixth Anniversary Meeting of the Provincial Medical and Surgical Association, held at Bath, July 18th and 19th, 1838.—By Jonas Malden, M.D. 3. Medical Topography of Copenhagen.—By C. Otto, M.D. 4. On the Medical Topography, Statistics, Climatology, and Natural History of Swansea, Glamorganshire.—By J. W. G. Gutch, Esq. M.R.C.S. 5. On the Medical Topography of Exeter and the Neighbourhood, being a Sketch of the Geology, Climate, Natural Productions, and Statistics of that District.—By Thomas Shapter, M.D. 6. On a Peculiar Affection of the Uvula.—By Edward Thompson, Esq. 7. A Report on Midwifery in Private Practice, with Observations on the Use of Ergot, &c. —By Caleb Burrell Rose, Esq. 8. Cases of Hernia Cerebri, with Remarks. —By G. Mallett, Esq. 9. A Case of Intus-Susceptio, with Separation of five inches of Intestine, followed by complete Recovery.—By John Fox, Esq. 10. A Case of Molluscum.—By C. Fowler, Esq. 11. On the Physiology of the Brain as the Organ of the Mind.—By Charles Cowan, M.D. E. and P. 12. Remarks on the Use of the Fucus Esculentus as a Bougie. —By Thomas J. Aitkin, M.D. 13. Observations on the Physiology and Function of the Liver.—By James Mc Cabe, M.D. 14. A Report of the Out-Patients attended by F. Ryland, Esq., at the Birmingham Town Infirmary, between the 25th December, 1836, and the 25th December, 1837. 15. A Report of the Cases of Sickness that occurred at the Asylum for the Children of the Poor of the Parish of Birmingham, between June the 30th and December 31st, 1837.—By F. Ryland, Esq. 16. Biographical Memoir of the late Henry Park, Esq. 17. Biographical Memoir of the late Nathan Drake, M.D.

Of many of these papers we can only speak in terms of merited praise. Need we say, that Dr. Boisragon delivered an eloquent address at Bath—that Dr. Malden took an able retrospect of medical matters and medical science—that the topographical articles display the indefatigable industry, and the careful judgment of their authors? If we do say as much, we but respond to the opinion, and echo the voice of every member of the Association. But excellent as these papers are, we are precluded, by the nature of this Journal, from doing more than notice them in terms of approbation.

The "Essays and Cases" are more adapted for our purpose, and we shall cast a glance at most of them.

### I. On a peculiar Affection of the Uvula. By Edward Thompson, Esq. Surgeon to the Whitehaven Infirmary.

Mr. Thompson is of opinion, that when, in simple inflammation of the throat, there is a sudden fatal termination, unpreceded by any dangerous symptom, enlarged uvula in all cases will be found to be the cause of death.

The affection, which Mr. Thompson intends to describe, consists in the elongation of the mucous coat of the uvula, by the gradual effusion of lymph, to such an extent as to endanger life, by its being inspired into the glottis, thereby causing spasm, and putting an immediate stop to respiration. The extent to which the elongation takes place occasionally is remarkable; he has seen it above two inches in length. The fluid contained in the expansion of the mucous coat is of a light amber colour, and, along with the membrane, so pellucid that the handle of a tea-spoon can readily be seen through it when placed behind the appendage. The body of the uvula appears, on examination, to be enlarged to a slight degree, partaking of the inflammation which extends across the palatine arch. He has not seen the tonsils enlarged.

The symptoms assigned to this alteration are the following:—

"The symptoms are those of simple cynanche, in the first instance; but afterwards a huskiness of voice, with an occasional brief loss of it, are superadded. These do not long continue in the same state, for as the disease advances, which, from what I have witnessed, it does very rapidly, another and more awful symptom arises—it is that of a distressful feeling of suffocation, perhaps slight at first, but which increases, and cannot, as at the commencement, be relieved by swallowing fluid. A superficial examination shows the throat inflamed, and, with the exception of the uvula, not much swelled. The latter may be seen slightly enlarged, but apparently of its usual length, which the hasty enquirer, judging by the red termination of it, may be easily deceived in. If death does not take place before he has made himself acquainted with the nature of the complaint, he may think the larynx is inflamed, or, which is more probable, œdema of the glottis to be present. Of the former, an examination of the symptoms will enable him to judge; of the latter, the finger will inform him of its non-existence. The frequent act of swallowing, carrying back as it does the flexible appendage into the œsophagus, is the cause of deception in this interesting yet dangerous affection, and is one reason of the great freedom, for a time together, from any distressing symptom, but which becomes suddenly changed by a cough and rapid inspiration. The position by such act is immediately altered, and the appendage may be instantly puked at it were into the larynx. The consequences of such a change may be at once imagined. The patient whom we had left with every assurance of a rapid recovery, may be instantly destroyed, or placed in a situation of the greatest hazard." 309.

Unfortunately Mr. Thompson has no case and dissection to bring forward in proof of the preceding statements. This is to be regretted. It is equally improper and impossible to attach more importance to the whole description than a clever suggestion may claim. We own that, for several reasons, we are not quite convinced.

Mr. Thompson relates two cases in illustration of his statements. We shall notice one.

*Case.* On the 24th November, 1834, Mr. T. visited Mr. H. He had complained of soreness in the throat during the night, which had rapidly got worse, and was accompanied occasionally with a slight feeling of suffocation, but this was quickly relieved by drinking water.

"On examining (I must acknowledge but imperfectly) the fauces, they were found inflamed, and the tonsils slightly swelled; the uvula was broader and apparently longer than natural, and highly injected. I thought I discovered its termination. He was directed a blister to the neck, and a gargle to be frequently used along with a purgative medicine, and told he would soon recover. I had not left the house half an hour before I was again hastily summoned, he having been attacked with such difficulty of respiration, and symptoms of suffocation, as to alarm the house. On arriving I found him as pale as a corpse, but then perfectly relieved in his breathing. He described his sensations during the paroxysm to have been most appalling, and that he was only saved from instant death by his wife beating him on the back, which excited cough, by which he was relieved immediately. Having seen a case somewhat similar, I was at once satisfied of the nature of the affection, and immediately blamed myself for not having more particularly examined the uvula. By pressing down the tongue I could see the commencement of the appendage, and on desiring him to cough, it was raised up, and brought forward by the spoon. The extended membrane reached to the front teeth, which enabled me to seize it and cut it off with a lancet. He was instantly relieved and suffered no more. The capsule containing the fluid which escaped into the mouth on the lancet being used, was formed of an extension of the mucous coat, extremely thin, and very diaphanous." 314.

We would direct our readers' attention to this case, as well as to the remarks of Mr. Thompson introducing it.

### II. A REPORT ON MIDWIFERY IN PRIVATE PRACTICE, WITH OBSERVATIONS ON THE USE OF ERGOT, &c. By CALEB BURRELL ROSE, Esq. Swaffham, Norfolk.

This Report occupies twenty-nine pages, and a very interesting, indeed instructive, one it is. We trust that others will imitate the excellent example set them by the gentleman before us, and that we shall have reports from private as well as from hospital practice. We cannot pretend to analyse Mr. Rose's paper, but must satisfy ourselves with picking out such parts as are most important.

*Dilatation of the Os Uteri in Lingering Labour.*—We are almost afraid to cite the sentiments of Mr. Rose on this head, militating as they do against those of that most pugnacious of men, Dr. Hamilton. He has inflicted on us a pamphlet already, and we could not well survive a repetition of the dose. But we will venture.

Mr. Rose alludes to Dr. Hamilton's advice, to endeavour to abridge the process of labour, by expediting its first stage with digital manipulation, not suffering it to extend beyond "twelve or fourteen hours, whenever the uterine contractions continue to be regular and progressive."

"Believing," he says, "the advice to be pregnant with mischief, if not positively dangerous, and having found that unfavourable consequences are to be attributed less to the *duration* of the first stage of labour, than to the *means* by which the dilatation is effected, I think it my duty to raise my humble voice in reprehension of the practice. Who is there amongst us that has not witnessed the ill consequences of officious meddling in the parturient process, manifested by the heat, dryness, and swelling of the vagina and os externum? In and *with* the doctor's *hands*, I grant, that such results would not be met with; but can we calculate upon such an immunity where the anxious and inexperienced youth has the managing hand? Do we not always desire that the opening of the mouth of the womb should be effected by the soft membranes distended by and protruded with the waters, rather than by the hard and cumbrous head? And why is it our desire? because experience has taught us, that when the membranes burst early, the labour is almost invariably more painful and tedious, and that *painful* interference may induce unmanageable effects. Here let me state a fact bearing strongly on this question; it is this, that in three severe cases out of four of hysteritis in my practice, the membranes had ruptured at the commencement of labour, and consequently the os uteri had been dilated by the head of the child. I may mention in this place also, that the only case of puerperal peritonitis that occurred among my patients, was preceded by the like untoward circumstance. These cases have contributed greatly to impress me with the belief in the pernicious tendency of the practice of manual dilatation." 321.

*The Ergot.*—This was used in ten cases, and, upon the whole, advantageously; in four instances it failed of its purpose; in two of the four it was given in the form of infusion, and, perhaps, was old and inert. Mr. Rose, on the whole, prefers Bass's Essence, as it sits easier on the stomach. The time required to accomplish its purpose, in those cases in which it was effective, has been from half an hour to an hour and a half.

Mr. Rose alludes to the opinions of Mr. Chavasse, that the ergot "is calculated, when given without care and discrimination, not only to defeat the purpose for which it was administered, but to destroy the vitality of the child in utero; and that, at other times, even when given under the guidance of judgment and discretion, it will occasion hour-glass contraction and retention of the placenta."

Mr. Rose thinks that the still-births seen after the exhibition of the ergot are probably the consequence of the nature of the uterine contractions excited by it—contractions almost continuous and unremitting, and most unlike the alternating action and repose of the uterus as it acts spontaneously. Under the influence of the ergot, the fœtal head is subject to a persisting force, instead of an alternating one, and driven like a missile, though with less velocity, through the unyielding pelvis, instead of advancing step by step, from the natural throes of labour; consequently, the brain is exposed to a degree of pressure incompatible with life.

As a consequence of this view, Mr. Rose conceives that we ought not to employ the ergot, till the hazard of long-continued pressure is passed by—in short, until the os uteri is fully dilated, and the head of the child occupies the *cavity* of the pelvis.

"In my own practice," he adds, "I have almost invariably employed the ergot as a mild substitute for the short forceps, and have administered it accordingly. I will give a case or two in illustration:—Mrs. B., thirty-two years of age, first pregnancy, membranes burst on a Saturday evening, trifling and irregular pains came on during that night and Sunday, in the evening pains more severe ensued at irregular intervals. I gave an anodyne. Monday morning I examined and found the os uteri but little opened, and the head presenting; at night the os uteri was not larger than a shilling. I gave an anodyne. Tuesday morning, os uteri the same. I bled her to sixteen ounces. In the evening, pains more regular, os uteri as large as a half-crown. I administered a domestic enema. Wednesday, two o'clock, A.M., os uteri fully dilated, and head entering the pelvis, pains inefficient, external parts still very rigid. I fomented them with warm water, and lubricated them internally with an abundance of lard. At eight o'clock, A.M., an ear could be felt under the pubis, and as the pains were declining, I gave half a drachm of ergot infused in two ounces of boiling water, repeating the dose every quarter of an hour; after the second dose, the action of the uterus was aroused, the third dose increased effectively the pains, and a living child was born in two hours; the placenta was expelled soon after." 326.

We leave these views to our obstetric brethren.

*Convulsions*.—In Mr. Rose's own practice, two cases exhibiting the premonitory symptoms of *convulsions* occurred. In both instances the unpleasant symptoms were completely removed by one large bleeding; they were first pregnancies. Mr. R. has been called into consultation in four instances within the last seven years. The first case turned out badly, and as Mr. Rose admits that it was injudiciously managed, we need say no more about it. In the second case, the patient was bled, first to twenty, then to sixteen ounces, and the forceps were used; she recovered. The third case was fatal, Mr. R. being only called in when the patient was evidently sinking. The fourth case proved fortunate. The patient was bled to thirty ounces, with but little benefit Then, says our author, "finding on examination the lower extremity of a child in the vagina, we proceeded to delivery, which was soon accomplished. Upon the birth of the child, we ascertained there was a second. The woman was perfectly unconscious of what was going on; as the uterine action was profitable, and the presentation natural, we left the birth of the second child to Nature, which she soon effected. The placentæ were readily yet not precipitately removed, and we were congratulating ourselves on having thus far proceeded safely, when a terrific paroxysm arose, and continued some seconds. Having protected the brain by the early detraction of blood, I advised fifty drops of Batley's Sedative Solution; it was presently given; mustard cataplasms were also applied to the feet. The patient soon settled into a quiet sleep, had no return of the convulsions, and, in short, did remarkably well."

*Hæmorrhage.*—"In the six hundred cases reported, *twenty* were complicated with hæmorrhage; of these, in *nine* the hæmorrhage occurred after the placenta was expelled, in *four* in the third stage of labour, and in *seven* before the birth of the child, of which two were *unavoidable* and five *accidental.* I shall merely observe on the thirteen cases comprising the first two classes of hæmorrhages, that the usual means for its suppression were employed with success; in two of these cases the placenta adhered partially, requiring the introduction of the hand to effect its separation; there was also a case of disruption, in which the hæmorrhage was excessive, requiring brandy in liberal doses; I also administered the ergot with decidedly good effect; indeed, latterly, I have invariably given the ergot in hæmorrhages occurring in the third stage of labour, but I prefer removing the placenta previously to administering it." 336.

We conclude our notice of this interesting Report, and turn to—

### III. Cases of Hernia Cerebri, with Remarks. By G. Mallet, Esq. Bolton.

Mr. Mallet relates five cases, and very interesting ones they are. Four of them were instances of hernia cerebri, all of compound fracture of the cranium. Every one did well—rare fortune or merit.

Mr. Mallett observes:—

"It will be seen that four of the five cases above-mentioned were affected with hernia cerebri, and that in each of these four cases a foreign body was found acting as a constant source of irritation, and knowing how generally the occurrence of irritating bodies found in other parts of the system is followed by excrescences, partaking more or less of the nature of the part from which they proceed, I think it extremely probable that without some such irritating cause, we should not have hernia cerebri, which opinion is much strengthened by the other case, in which the complaint did not come on, as no broken and loose portion of bone, or any other extraneous particles, were found at any period of the progress of the case after the first dressing." 350.

Mr. M., at the same time, protests against being understood to affirm that hernia cerebri cannot occur without the presence of an irritating body—a necessary caution, for few would be got to accede to such a bold hypothesis. Yet he believes, and would direct attention to the probability of the circumstance, that there is a connexion between the two. He therefore advises that we cannot be too careful in removing, not only all the parts perfectly loose, but also all the points or spiculæ of bone that may still be adherent to the cranium, if they are likely to press upon the brain; and he would also recommend that the finger be gently pressed into the wound, so as to examine into the state of the interior table of the skull, and if any portions of it appear so cracked or broken, that it is probable they will exfoliate and be thrown off, if not removed, it is better that they should be taken away, even if some slight violence be necessary to extract them.

We leave this advice to our surgical readers. It does appear to sin against the ideas which at present prevail. We say *ideas*, for how uncertain and vague are our opinions on the treatment of severe injuries of the cranium!

The following are Mr. Mallett's sentiments on the best method of manag-ing hernia cerebri :—

" In every case when the hernia first commenced, moderate pressure and various topical applications were used, but in two of the cases they were found totally inefficient to restrain the progress of the complaint; and in the case of Howcroft it increased to the enormous magnitude mentioned. It was then re-moved, and the result is a satisfactory proof that, in cases of necessity, the mass, no matter how large, may be at once cut off with safety and advantage. After the operation, let the wound be carefully examined, to be certain that there is no irritating cause remaining likely to bring on its reproduction. If, after this, there should be a disposition to its reproduction, moderate pressure and the daily application of the nitric acid, reduced to the following strength, will be the best applications :—℞. *Acidi Nitrici* ♏. xl., *Aquæ fontanæ* f. ℥ij., *Fiat Lotio.* I am unable to assign any particular reason why the above application should be more effective than any other, but the result after each application decidedly proved it to be so." 351.

Mr. Mallett gives a dissection of a large hernia cerebri sliced off by him, which will be perused with instruction. The appearance, he states, of the cut part upon the head was that of medullary matter surrounded by its cine-ritious or cortical envelope, and of course the corresponding section upon the tumor was similar. The exterior of the tumor was formed by a very thin layer of granulations of a brownish colour. A vertical section being made through the mass, it exhibited throughout, immediately under the above-mentioned thin granular covering, a stratum of the cineritious matter, nearly half an inch in thickness, where it was cut from the head, and it became gradually attenuated as it receded from that part, till at the most distant of highest point it was so thin as to be scarcely perceptible. The remainder of the tumor so resembled the medullary structure of the brain, that no eye could detect the one from the other; the only difference being, that it was not quite so firm in structure as the brain usually is. In the centre, a cavity was laid open by the section, containing about an ounce of a limpid serum; the cavity was lined throughout by a beautiful transparent and glistening membrane, and if the shape had not been different, it might have been mis-taken for one of the ventricles of the brain. He therefore regards the hernia, not simply as consisting of fungous granulations, nor yet of an elevation of the brain previously contained within the skull, but as a new formation similar to brain.

### IV. A Case of Intus-susceptio, with separation of Five Inches of Intestine, followed by complete recovery. By John Fox, Esq. Surgeon, Cerne Abbas.

H. Diment, aged 16, ate some nuts on the 8th September, felt unwell on the 9th, and was seen by Mr. Fox on the 10th. He had pain about the umbilicus, and had had no motion for twenty-four hours. It is unnecessary for us to particularise symptoms or treatment, suffice it to say that the case assumed all the characters of intus-susceptio, and resisted all the remedies directed against that disease by Mr. Fox. But on the 16th, it occurred to Mr. F. to give a trial to inflation. He immediately procured a bladder, and secured one end of it to the nozzle of a pair of bellows, and the other end to a common enema pipe, and having introduced the pipe its full length into the rectum, the bellows were set in motion by his pupil, and inflation forcibly but slowly persevered in for many minutes, until the poor boy complained of a disposition to "break wind," and said that his "belly was very tight." The convolutions of the intestines could be seen and felt distinctly, the arch of the colon most so; the tube was now withdrawn, and, to Mr. F.'s great surprise and gratification, in about twenty minutes he said he felt as if he should soon have a stool; he was therefore lifted and supported upon the bedpan, when he passed off wind in large quantities, which in a few minutes was followed by a very copious and liquid evacuation, containing, however, a few hard lumps. He continued, after this, to pass flatus and stools, and to improve, until the 22nd, when he was decidedly worse. Mr. Fox left him, supposing the case hopeless. On his arrival next morning, he was agreeably disappointed. He found that:—

"My patient's bowels had been very copiously moved, that he had afterwards slept for nearly two hours, and that he expressed himself as feeling in every respect more comfortable; at the same time, however, his nurse told me she 'supposed he would not last many days longer, as a large piece of his bowels had come away with one of his stools.' I immediately examined the stool, removed the substance alluded to, and having carefully washed it, I found that the woman's suspicions were correct, and that it was indeed a portion of one of the intestines, with some of the mesentery still adhering." 362.

The lad recovered perfectly. Mr. Fox naturally regrets that he did not resort to inflation earlier.

### V. A Case of Molluscum. By C. Fowler, Esq., Surgeon to the Cheltenham Dispensary and Casualty Hospital.

The subject of this well-marked case, W. Jones, aged 68, is a day labourer. He stated himself to have been one of a family of nineteen,—never to have contracted any syphilitic complaint, or suffered from any kind of disease, hereditary or otherwise.

"This singular affection, he states, first made its appearance about twenty or thirty years since, commencing in small accuminated elevations upon the waist and wrists, and gradually increasing to their present magnitude. The large and pendulous tumor hanging from the crest of the left ilium, and which it was customary with him to place in his breeches pocket for support whilst at work, was the first that made its appearance; this, and another large one between the arm and thorax of the same side, were the only ones which, from occasional subjection to pressure, gave him any degree of uneasiness or pain. These tumors were thickly disseminated over the whole cutaneous surface, from the crown of the head to the sole of the foot, and varied in size from the smallest visible elevation of the skin, to the one seen on the left waist, which weighed about three quarters of a pound; some of them were attached by broad bases and others by stems of various degrees of length and magnitude. The tumors upon the back and waist were the most thickly set of any, and looked like clusters of reddish grapes; those upon the abdomen and hips were longer and more irregular in form. The skin covering them was perfectly sound, but possessing more of a rose tint than the surrounding integument; in those, however, that had been subjected to pressure upon their summits, the colour had changed to a dirty blueish grey, the skin likewise was very much attenuated, and easily raised raised from, and rolled over the subjacent tumors; forcible compression with the fingers seemed to produce very little uneasiness in them, and the sensation to the touch, when handled, very nearly resembled that of fatty tumors, only somewhat harder in consistence, and many of them, particularly the larger ones, were distinctly lobulated.

Two of these tumors, of moderate size, situated upon the abdomen, were removed for the purpose of analysing their structure; and on making a section of them, no very great difference was observable in them from the appearance of fatty tumors generally; there was the same semi-transparent yellowish tinge pervading the whole mass as in the latter, but the consistence was denser, the cut surface smoother and firmer, and the substance more uniform throughout. The skin was easily reflected from them in consequence of its loose connection, and a fine cellular membrane surrounded the whole, and dipped into the interstices of those that were lobulated. The excision of these tumors was accompanied with the loss of but a few drops of blood, which were evidently supplied by the surrounding integuments, for no nutrient vessel could be discovered entering at their base." 366.

One, and one only growth, on the crown of the head, contained atheromatous matter.

Mister Jones is making a tour through the great towns, to exhibit himself to the faculty. He is accompanied by an eminent professional authority, as showman—the hospital errand boy.

VI. THE FUCUS ESCULENTUS, AS A RECTUM BOUGIE. By THOMAS J. AITKIN, M.D. Poole, Dorsetshire.

Dr. Aitkin relates a case for the purpose of introducing the fucus esculentus (tangle) found abundantly on several of our rocky coasts, as an excellent rectum bougie. To mention the recommendation is enough. Dr. A. assures us that the fucus answers well.

There are several excellent memoirs, biographical and physiological, which we dismiss with reluctance, as not adapted for our pages. The volume before us is a good one.

